# MicroRNAs, Hepatitis C Virus, and HCV/HIV-1 Co-Infection: New Insights in Pathogenesis and Therapy

**DOI:** 10.3390/v4112485

**Published:** 2012-10-26

**Authors:** Archana Gupta, Gokul Swaminathan, Julio Martin-Garcia, Sonia Navas-Martin

**Affiliations:** 1 Department of Microbiology and Immunology, Drexel University College of Medicine, 245 North 15th Street, Philadelphia, PA 19102, USA; Email: ag434@drexel.edu (A.G.); gs337@drexel.edu (G.S.); julio.martin-garcia@drexelmed.edu (J.M.G.); 2 Microbiology and Immunology Graduate Program, Drexel University College of Medicine, 245 North 15th Street, Philadelphia, PA 19102, USA; 3 Center for Molecular Virology and Translational Neuroscience, Institute for Molecular Medicine and Infectious Disease, Drexel University College of Medicine, 245 North 15th Street, Philadelphia, PA 19102, USA; 4 Department of Laboratory Medicine, Veterans Affairs Medical Center, San Francisco, CA 94121, USA

**Keywords:** microRNA, miR-122, exosomes, HCV, hepatitis C virus, hepatitis, antiviral response, HIV-1, HIV-1/HCV co-infection, antagomir, therapeutics

## Abstract

MicroRNAs (miRNAs) can exert a profound effect on Hepatitis C virus (HCV) replication. The interaction of HCV with the highly liver-enriched miRNA, miR-122 represents one such unique example of viruses having evolved mechanism(s) to usurp the host miRNA machinery to support viral life cycle. Furthermore, HCV infection can also trigger changes in the cellular miRNA profile, which may ultimately contribute to the outcome of viral infection. Accumulating knowledge on HCV-host miRNA interactions has ultimately influenced the design of therapeutic interventions against chronic HCV infection. The importance of microRNA modulation in Human Immunodeficiency Virus (HIV-1) replication has been reported, albeit only in the context of HIV-1 mono-infection. The development of HCV infection is dramatically influenced during co-infection with HIV-1. Here, we review the current knowledge on miRNAs in HCV mono-infection. In addition, we discuss the potential role of some miRNAs, identified from the analyses of public data, in HCV/HIV-1 co-infection.

## 1. Introduction

**Figure 1:**
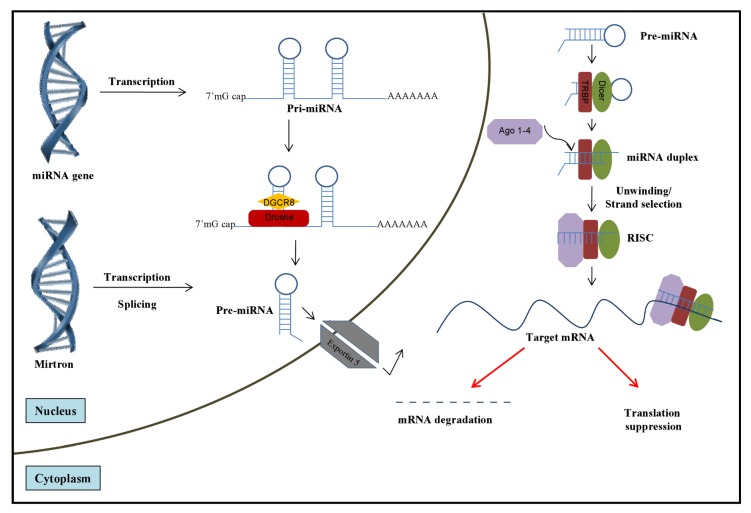
Biogenetic pathway of cellular miRNAs. MicroRNAs are transcribed from either independent miRNA genes as primary miRNAs (pri-miRNAs) or introns of protein coding genes (mirtrons). The pri-miRNA is cleaved by the Rnase III enzyme Drosha and its binding partner DGCR8 to a hairpin shaped precursor miRNA (pre-miRNA), which is recognized and exported by the nuclear export receptor Exportin 5 and Ran-GTP into the cytoplasm. Once in the cytoplasm, the pre-miRNA is processed by Dicer into a miRNA duplex which is then loaded onto Argonaute proteins to form a RNA-induced silencing complex (RISC). The fate of the target mRNA recognized by the RISC complex depends on the degree of complementarity between the miRNA and its cognate sequence on the mRNA. Typically, identical sequence match between the miRNA and its target site within the 3’ UTR of the transcript leads to mRNA cleavage and subsequent degradation while incomplete complementarity often results in suppression of translation.

MicroRNAs (miRNAs) are a novel class of small non-coding single-stranded RNAs that regulate gene expression at the posttranscriptional level by binding to target messenger RNAs (mRNAs) [1,2]. Although it is likely that not all of their functions have been fully identified, emerging evidence indicates that miRNAs play a major role in fundamental cellular processes, the regulation of the immune system, and in the onset and progression of many diseases [3,4,5]. Using *in silico* approaches it has been estimated that miRNAs regulate more than 60% of human protein-coding genes [6], and over 2,000 human mature miRNAs have been annotated (miRBase v19.0; http://www.mirbase.org/). They are transcribed in the nucleus by RNA polymerase II as primary miRNAs (pri-miRNA) that harbor the mature miRNA sequence within the stem of an imperfect ~80 nt hairpin RNA (reviewed in [7]). The pri-miRNA is processed by the microprocessor, consisting of nuclear RNAse III enzyme Drosha and the double stranded RNA-binding protein partner DiGeorge syndrome Critical Region 8 (DGCR8), into precursor miRNA (pre-miRNA) that is subsequently transported to the cytoplasm. The pre-miRNA is cleaved by the cytoplasmic enzyme Dicer into an imperfect 22 nucleotide RNA duplex characterized by two nucleotide 3’ overhangs at each end. Generally, the miRNA strand that exhibits weaker 5’ base pairing is preferentially loaded onto RNA-Induced Silencing Complex (RISC) that guides the recognition of partial matches, generally within the 3’ untranslated region (UTR) of mRNAs. The binding of miRNA to its cognate sequence on the mRNA leads to translational repression or enhanced mRNA degradation (Figure 1). Two independent recent studies have determined the kinetics of translational repression and mRNA decay and have found that miRNAs seem to first block translation of their mRNA target and, subsequently, to mediate its degradation [8,9], although whether this is a general mechanism remains to be demonstrated.

Interestingly, recent data support the notion that miRNAs are key players in virus-host interactions and viral pathogenesis [7,10,11,12,13,14,15,16]. The role of miRNAs in the complex regulatory network that controls both viral and host gene expression in the infected cell is starting to be elucidated for some pathogenic viruses. DNA viruses can encode their own miRNAs, and more that 225 viral miRNAs have been identified, although the function of only a few miRNAs has been demonstrated [17,18]. In contrast, the existence of viral miRNAs in RNA viruses is controversial. At least theoretically, the lack of access to nuclear miRNA processing machinery, and the destabilizing effects of miRNA processing on RNA genomes are major barriers that RNA viruses would need to overcome. Remarkably, and despite those barriers, retroviruses, a flavivirus, and influenza virus have been engineered to express biologically active miRNAs or miRNA-like oligonucleotides when a pre-miRNA sequence is incorporated into the viral genome [19,20,21]. These data suggest that viruses with RNA genomes can express miRNAs through Drosha-independent mechanisms. In support of this hypothesis, Hussain, *et al.,* [22] have identified a miRNA-like small RNA in the 3’UTR of West Nile virus, which is produced during viral infection in mosquito cells and, remarkably, leads to an accumulation of GATA4 mRNA that facilitates virus replication. In addition, viral infections trigger changes in the cellular microRNAome that can modulate the expression of host proteins to the benefit of the virus. For example, Hepatitis C virus (HCV) infection enhances miR-130a expression, which in turn inhibits endogenous Interferon-induced transmembrane protein 1 (IFITM1) expression in a hepatoma cell line [23]. Furthermore, cellular miRNAs can target and repress the expression of viral mRNAs [24,25,26]. Although there are some examples on how cellular miRNAs can stimulate virus replication through indirect or unknown mechanisms [27,28], at least one cellular miRNA (miR-122) facilitates viral infection (HCV) through direct target of the 5’UTR of the viral genome [29,30].

Hepatitis C virus (HCV) infection is the leading cause of chronic hepatitis, liver cirrhosis and hepatocellular carcinoma affecting 180 million people worldwide [31]. Currently, there are no protective vaccines against HCV. Although acute HCV infection resolves spontaneously in some patients [32], persistent infection with chronic liver disease develops in more than 70% of patients, of whom approximately 20% will develop cirrhosis [33]. The present standard of care, a combination of pegylated interferon (Peg-IFN)-α and ribavirin, is suboptimal and sustained virological response is achieved only in about 50% of patients (depending on the viral genotype) and the treatment is associated with several side-effects, some of which can be severe [34]. After more than two decades in which no new antivirals against HCV had been developed, two NS3/4A protease inhibitors, Telaprevir and Boceprevir, were approved by the FDA earlier this year. The drugs have improved response rates, however because they need to be administered with the standard treatment to achieve sustained viral clearance, the therapy is associated with a higher risk of adverse events [35,36]. The search for an interferon-free regimen and the discovery of the remarkable role of miR-122 in the HCV viral cycle has paved the way for a novel class of drugs called antagomirs, which silence endogenous microRNAs, to be investigated as a therapeutic option against chronic HCV infections [37,38]. The specific inhibitor of miR-122 (Miravirsen, a β-D-oxy-Locked Nucleic Acid (LNA)-modified phosphorothioate anti-sense oligonucleotide; Santaris Pharma A/S) is the first miRNA-targeting treatment to enter into human clinical trials, and it is currently in phase 2 for the treatment of HCV infection [38].

Activation of the innate immune response through Toll-Like Receptors (TLRs) has a profound impact on the cellular microRNAome. Several miRNAs have been shown to be up-regulated in response to TLR ligands, and many directly target components of the TLR signaling system, and downstream effectors such as interferons (IFNs), cytokines and chemokines (reviewed in [39,40]). Human Immunodeficiency Virus (HIV-1) infection of macrophages plays a key role in viral pathogenesis and progression to AIDS. Interestingly, our group has provided evidence that miR-155, a key regulator of inflammatory and immune responses, exerts an anti-HIV-1 effect by targeting several HIV-1 dependency factors involved in post-entry and pre-integration events, leading to severely diminished HIV-1 infection of macrophages [41]. Our studies provide evidence of novel microRNA-155 targets and may serve as the basis for innovative approaches to reduce cellular susceptibility to HIV-1 infection.

Due to their similar routes of transmission (sexual and blood-borne), co-infection of HIV-1 and HCV is relatively common. Of the 35 million people infected with HIV-1 worldwide, around 20% (seven million) had chronic hepatitis C [42]. This population is mainly represented by individuals with a past history of intravenous drug use, although frequent outbreaks of HCV among homosexual men have been reported in the last decade [43]. The effects of HIV-1 on the pathogenesis of HCV infection include increased viral loads, a higher rate of viral persistence, a faster rate of fibrosis progression, and higher rates of hepatic decompensation [44,45,46]. The reciprocal effects of HCV on HIV-1 disease progression are less clear. Based on our current understanding, the major contribution of HCV to mortality of HIV-1 co-infected individuals is attributable to accelerated liver disease and not to increased AIDS-related complications [47,48].

In this review, we focus on the dynamic interactions between HCV and the host miRNA pathway and its effect on disease pathogenesis. In addition, based on data from the literature on HCV and HIV-1 mono-infection, we have identified some miRNAs of interest in the setting of HCV-HIV-1 co-infection. We discuss their potential role in co-infection with HCV and HIV-1 viruses.

## 2. Role of miRNAs in HCV Infection

### 2.1. Role of Cellular miRNAs in Regulating HCV Infection

HCV is a single stranded, positive sense RNA virus and its 9.6 kb genome encodes a precursor polyprotein that is approximately 3000 amino acids in length and is co- and post-translationally processed by viral and cellular proteases into four structural proteins (core, envelope proteins E1, E2, and the viroporin p7) and six non-structural viral proteins (NS2, NS3, NS4A, NS4B, NS5A, and NS5B) (reviewed in [49]). The genome is flanked by 5’ and 3’-UTRs with extensive secondary structures that are essential for viral replication, translation, and likely for encapsidation [49]. In contrast to cellular mRNAs, HCV translation is cap-independent and mediated by an internal ribosome entry site (IRES) located within the 5’UTR. The structural proteins include the core protein, which forms the viral capsid, while the E1 and E2 glycoproteins mediate viral entry and fusion. The non-structural proteins are required for RNA replication and are cleaved from the HCV polyprotein by NS2 and NS3 viral proteases. HCV circulates *in vivo* as a dynamic distribution of divergent but closely related genomes (quasispecies) subjected to a continuous process of genetic variation, competition, and selection [50]. Eleven major HCV genotypes with several subtypes have been identified on the basis of phylogenetic analysis [51].

Although mammalian miRNAs may not constitute a *bona fide* cellular antiviral response, in several instances, they can have a profound suppressive effect on the replication of several pathogenic viruses. Considering that the IFN system is a key component of the innate immune defense against viral infections [52], the possibility for IFNs to mediate its effect, at least partially through induction of miRNAs is interesting. Indeed, a microarray analysis of RNA derived from IFN α/ β or IFN γ stimulated cells identified ~30 miRNAs that were differentially modulated in response to the treatment [53]. Remarkably, out of the 30 miRNAs, eight exhibited nearly perfect complementarity in their seed sequences to the HCV genome. Functional studies on the relevant miRNAs revealed that over-expression of miR-196, -296, -351, -431 and -448 led to a substantial inhibition of HCV replication in a human hepatoma cell line, indicating that the miRNAs had an antiviral effect against the virus. Furthermore, miR-196 and -448 were shown to exert the antiviral effect by directly interacting with the NS5A and core sequences of the viral RNA respectively [53].

Subsequently, using a bioinformatics approach, Murakami and colleagues identified that miR-199a-3p has a seed sequence match within domain II of the HCV IRES, which is conserved across all HCV genotypes [54]. Exogenous expression of the miR-199a-3p markedly restricted HCV replication in liver cells harboring the full genomic replicon of HCV genotypes 1b and 2a. The effect was shown to be independent of IFN induction and primarily driven by direct interaction between the viral and miRNA sequences. Bioinformatics analyses have been used to identified let-7b as a new regulator of HCV replication [55]. In particular, let-7b suppressed HCV replicon activity and down-regulated HCV accumulation leading to reduced infectivity of cell culture-derived HCV (HCVcc). Mutational analysis identified let-7b binding sites at the coding sequences of NS5B and 5'-UTR of HCV genome that were conserved among various HCV genotypes. The underlying mechanism for let-7b-mediated suppression of HCV RNA accumulation is not completely understood but it seems to be independent of inhibition of HCV translation [55].

In addition to targeting the viral RNA directly, miRNAs can also exert an antiviral effect by modulating (directly or indirectly) the expression of cellular genes that suppress or facilitate viral replication. Heme oxygenase 1 (HMOX1), a cytoprotective protein with anti-inflammatory and antioxidant properties is negatively regulated by Bach1, a basic leucine zipper mammalian transcriptional repressor [56]. Both the HMOX1 mRNA as well as protein levels are found to be suppressed in livers of chronically infected HCV patients [57]. However, over-expression of HMOX1 attenuates HCV replication suggesting that the enzyme may play an important role in inducing a potent anti-HCV response [58]. Hou *et al.,* demonstrated that the Bach1 mRNA has two seed region matches of miR-196 in its 3’UTR and over-expression of the miRNA leads to significant suppression of the Bach1 protein and consequently, up-regulation of HMOX1 and down-regulation of HCV [25]. Therefore, the antiviral mechanism of miR-196 against HCV seems to be through a combination of the inhibitory effect on Bach1 expression and the recognition of the viral genome by the miRNA. 

The fact that several miRNAs can suppress HCV replication either through interaction with the HCV RNA or by modulating host factors that play a role in the viral life cycle may explain tropism restriction of the virus due to tissue specific miRNA content. Based on the literature, at least three and five miRNAs have been shown to promote HCV infection or have “anti-viral” effect (summarized in Table 1 and Table 2, respectively).

**Table 1:** MicroRNAs that enhance Hepatitis C virus (HCV) infection

microRNA	Mechanism	Target on HCV	Target on HCV-infected cells	Other effects in host and targets
miR-122	Binding to HCV; Enhanced translation [29,59,60,61]	5’UTR	Independent	Liver homeostasis; Tumor suppressor; Lipid metabolism; Anti-inflammatory [62,63,64]
miR-491	Inhibition of the PI3K/Akt pathway [27]	NR	PI3K/Akt pathway	Apoptosis; Bcl-X(L) [65]
miR-141	Inhibition of tumor-suppressor DCL-1[66]	NR	DCL-1[66]	Cancer; oxidative stress response [67]
				Anti-HBV through peroxisome proliferator-activated receptor alpha (PPARA) [68]

Note: NR, not reported

**Table 2:** MicroRNAs that inhibit HCV infection

microRNA	Relationship with IFN	Mechanism	Target on HCV	Target on HCV-infected cells	Antiviral effect on other viruses
miR-199a-3p	Independent of IFN [54]	Binding to HCV, [54]	5’UTR IRES [54]	NR	HBV [24]; Herpesvirus and SFV [25]
miR-196	Induced by IFN [53]	Binding to HCV and host protein [69]	NS5A	Batch1[69]	NR
miR-29	Induced by IFN [53]	NR [70]	NR	Extracellular matrix proteins [70]	Influenza [26]; HIV-1 [71]
miR-let-7b	Regulates IFNβ expression [72]	Binding to HCV, no effects on translation [55]	5’UTR, NS5B [55]	NR	NR
miRs-296,-351, -431, and -448	Induced by IFN [53]	NR[53]	NR	NR	NR

Note: NR, not reported

#### 2.1.1. MicroRNA-122: A Master Regulator of HCV Infection?

The interaction of miR-122 with the HCV RNA is a unique example of how viruses have evolved strategies to usurp the host miRNA biosynthetic machinery to propagate viral replication (reviewed in [59]). MicroRNA-122 is highly liver-specific and accounts for ~70% of the total miRNA content of the liver [73]. The physiological role of miR-122 was revealed by *in vivo* silencing of the miRNA via intraperitoneal administration of 2’-O-methoxyethyl phosphorothioate modified antisense oligonucleotide in mice [74]. Inhibition of cellular miR-122 resulted in the alteration of several genes involved in fatty acid and cholesterol biosynthesis indicating that the miRNA plays a significant role in regulating lipid metabolism in the liver. The cellular function of miR-122 was also confirmed in non-human primates using a locked- nucleic-acid modified oligonucleotides (LNA-antimiRs) platform to silence the endogenous miRNA [75]. Importantly, in comparison to the 2’-O-methyl modified oligonucleotides, LNA-antimiRs exhibited a stronger suppressive effect on HCV expression in human hepatoma cells harboring an HCV replicon. 

**Figure 2:**
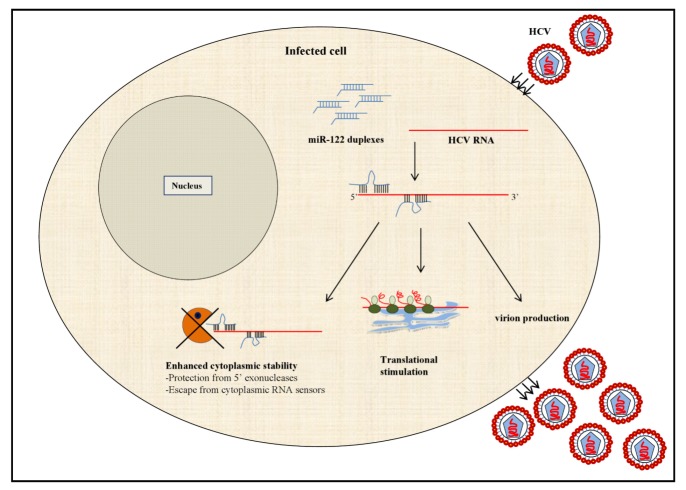
Mechanisms implicated in miR-122 mediated increase in HCV RNA abundance. MicroRNA (miRNA)-122 binds at two sites within the 5’UTR of the HCV RNA forming a unique miRNA-viral RNA complex characterized by 3’ overhang and internal bulge within the miRNA molecules. The 3’ overhang of miR-122 at the first site is proposed to mask the 5’ end of the HCV RNA and to increase its cytoplasmic stability by preventing its recognition by cytoplasmic RNA sensors such as the retinoic acid-inducible gene-I (RIG-I) as well as nucleolytic digestion by 5’ exonucleases. Additionally, binding of miR-122 to the HCV RNA stimulates virus release and viral RNA translation by enhancing the association of the 48S translation initiation complex.

The dependence of HCV on hepatic miR-122 for viral replication was first demonstrated by Jopling *et al.,* in 2005 [29]. The investigation was prompted from the observation that HepG2, a human liver derived cell line that weakly supports HCV replication, lacks miR-122 expression while liver tissue and other hepatoma cell lines that allow robust viral replication express substantial levels of the miRNA. The study showed that miR-122 binds at two sites, S1 and S2, in the 5’UTR of the HCV RNA and is required to maintain viral abundance in the infected cell. Further analysis revealed that miR-122 mediated amplification of HCV RNA is at least in part due to an increase in viral RNA translation and infectious virus production. However, while both S1 and S2 sites contribute equally to enhancing translation, S1 outweighs the latter in regulating the virus yield [30]. The increase in translation by miR-122 occurs by accelerated association of the small ribosomal subunit in the 48S complex during the initiation phase [76]. Furthermore, it has been shown that miR-122 mediated translational enhancement is a specialized process that requires uncapped mRNA, the IRES of the HCV RNA and the 3’ region of the miRNA [77]. Thus, the prerequisites for miR-122 to stimulate translation offer a possible explanation to why the observed phenomenon is so unique (Figure 2).

Apart from stimulating translation and virion release, increased stability of the viral genome has also been implicated as one of the mechanisms by which miR-122 regulates HCV RNA abundance [78]. Unlike typical miRNA-mRNA interactions, which require the binding of the miRNA seed sequence to its cognate target on the RNA molecule, miR-122 binds to the HCV RNA at several internal sites to exert its effect. The internal nucleotides in each of the miR-122 sites bind to the viral genome and form a bulge while the 3’ nucleotides interact with the 5’ end of the HCV RNA creating a 3’ over-hang. The formation of this unique physical complex formed between the miRNA and HCV is proposed to prevent the viral RNA from being recognized by cytoplasmic sensors or undergo nucleolytic degradation [78].

In view of the role of miR-122 in the HCV life cycle, the observation that certain non-hepatic cell types lacking miR-122 expression can support HCV replication presents a challenge in understanding the requirement of the miRNA for the virus. Chang and colleagues addressed this question by exogenously expressing miR-122 in non-hepatic cells harboring a sub-genomic replicon of HCV and observed a substantial increase in HCV replication suggesting that although miR-122 is not an absolute requirement for the virus, it can significantly enhance viral accumulation [79]. A similar observation was also made in HepG2 cells wherein miR-122 expression allowed the cells to support efficient HCV RNA replication as well as infectious virion production [80].

MicroRNA duplexes typically associate with Argonaute proteins (Ago 1-4) in the pre-RISC complex where they are unwound and separated into guide and passenger strand. The passenger strand is discarded while the guide strand along with the Ago protein forms the mature RISC that mediates translational repression. Considering that miR-122 enhances HCV translation rather than repressing it, an obvious question that arises is whether miR-122 requires the canonical RISC complex to mediate its effect on HCV RNA. Indeed, miR-122 requires Ago2 to enhance HCV translation and accumulation [81]. Depletion of cellular Ago2 in human hepatoma cells reduces HCV RNA levels suggesting that miR-122 binds to an Ago2 containing complex to exert its effect. Alternatively, miR-122 and Ago2 may function separately such that Ago2 may simply be involved in unwinding the miR-122 duplex and delivering it to the 5’UTR sites on the viral RNA to exert its positive effect on viral translation.

Several proteins associated with the miRNA gene silencing complex are present in P bodies (reviewed in [82]). These cytoplasmic foci harbor translationally-silenced mRNAs and are equipped with enzymes required for mRNA deadenylation and degradation. In addition to P bodies, stress granules comprising of untranslated mRNAs, translation initiation factors and 40S ribosomal subunits also form in the cytoplasm in response to various stress conditions. Both P bodies and stress granules regulate mRNA translation and metabolism and consequently the proteins associated with the processes. Interestingly, HCV disrupts and relocates the components of P-bodies and stress granules to HCV replication sites near lipid droplets within the infected cell [83]. Furthermore, several miRNA effectors such as Dead Box RNA helicases 6 (DDX6) [84], ataxin-2 (ATX2), poly-A binding protein 1 (PABP1), and Sm-like protein (Lsm1) that constitute P bodies and stress granules positively regulate HCV replication possibly through interaction with miR-122 in the RISC complex [83].

Given its ability to profoundly impact the HCV life cycle, miR-122 is an attractive therapeutic target against chronic HCV infections. Early in 2010, Lanford *et al.,* published a proof-of-concept study demonstrating that administration of LNA modified oligonucleotide (SPC3649) against miR-122 led to prolonged suppression of HCV viremia in chronically infected chimpanzees [37]. The infected animals were administered weekly intravenous doses of the antagomir for 12 weeks and were subsequently monitored for a 17-week treatment-free period. A significant reduction in HCV viremia was observed three weeks into the treatment. Importantly, HCV viremia continued to be below the baseline up to 16 weeks following the discontinuation of the treatment, highlighting the stability and efficacy of the drug. A major setback that has prevented the success of several anti-HCV drugs has been the emergence of resistant strains leading to rapid viral rebound; however, deep sequencing of the viral strains recovered from the infected animals following treatment revealed that there was no accumulation of adaptive mutations in the two miR-122 binding sites. Additionally, a marked suppression in the interferon stimulated genes was observed in animals even without complete suppression of HCV viremia suggesting that the drug may have the ability to salvage IFN unresponsiveness frequently observed in non-responders during IFN therapy. Furthermore, no adverse effects were reported with the regimen.

Following the pre-clinical success against HCV of the compound SPC3649 (Miravirsen), Santaris Pharma A/S has now moved the drug to phase 2a clinical trials for evaluation of its safety, tolerability and efficacy in treatment naïve chronic HCV patients [38]. If successful, Miravirsen may circumvent the major limitations associated with the current standard therapy to treat HCV infection and offer hope to patients infected with viral strains that are resistant to IFN treatment. However, although to date these results are promising, it has been already suggested that the combination of various miRNAs together with miR-122 could be even more successful than antagonizing miR-122 alone [85].

### 2.2. HCV Modulation of the Cellular miRNAome

There is increasing evidence demonstrating changes in the expression of miRNAs in cells infected with viruses belonging to different families. Whether this deregulation is driven directly by viral factors or is a cellular response to infection itself is difficult to prove, and as previously noted, this difference is essentially semantic [86,87]. HCV infection has been shown to induce changes in the cellular miRNA expression profile that may arise as a result of direct interaction between viral factors and the miRNA machinery or a consequence of cellular response to the infection [88]. Numerous studies have investigated the miRNA alterations in different tissues of chronic HCV patients to identify markers that may help to correlate the miRNA changes to the stage of disease, to predict response to therapy, or to have a diagnostic value [89,90,91,92,93,94]. Several studies have identified mechanisms for individual deregulated miRNAs in HCV-infected cells with potential biological significance in infected patients [66,69,70,95,96]. Additionally, identification of deregulated miRNAs in HCV infected individuals may widen the group of miRNAs that can potentially be targeted through antagomir based therapy against the virus. For instance, the expression of miR-21, implicated in tumorigenesis [58,97] and cell proliferation [98,99] is significantly increased in hepatocytes of HCV-infected patients and correlates with viral load, fibrotic stage and serum levels of liver transaminases [100]. Interestingly, miR-122, known to enhance HCV replication is down-regulated in late stages of HCV-associated liver fibrosis [100]. Lower pre-treatment miR-122 expression in the liver has also been suggested as a predictor for HCV patients who will potentially fail interferon therapy [99]. In that regard, non-responders exhibit a pre-activated interferon phenotype in the liver such that administration of pegylated IFN does not alter the expression of IFN stimulated genes [101]. It is therefore likely that the expression of miR-122, which is inversely regulated by IFN, may be suppressed in hepatocytes due to endogenous activation of IFN pathways in the setting of a viral infection. While several studies have demonstrated a decrease in the hepatic miR-122 expression levels during chronic HCV infection, Varnholt *et al.,* have reported an aberrant over-expression of miR-122 as well as miR-100 and -10a by quantitative analysis of formalin-fixed paraffin-embedded samples of HCV induced primary liver tumors consisting of hepatocellular carcinomas (HCC) and premalignant dysplastic liver nodules [102]. The potential diagnostic and prognostic use of miRNAs in hepatitis C-related HCC is an emerging and rapidly expanding field [103,104,105]. The difference in miR-122 expression levels in the liver tissue of HCV infected patients observed by various groups suggests that the cellular miRNA environment constantly undergoes changes with progression in disease state. Another study investigating the miRNA expression profile of liver biopsies from HCV-infected patients has reported differential expression of a host of miRNAs related to antigen presentation (miR-105), immune response (miR-105 and -34c), cell cycle (miR-190), proteasome (miR-211 and -133b) and lipid metabolism (miR-134) during infection [106].

Chronic HCV infection can also induce miRNA changes in the cells of the immune system, which may ultimately contribute to rendering the immune cells dysfunctional, a phenomenon observed in patients who fail to clear the virus [107,108,109]. Scagnolari *et al.,* [110] examined the expression levels of miRNAs with IFN mediated antiviral effects against HCV in the peripheral blood mononuclear cells (PBMCs) of chronic HCV-infected individuals. While increased baseline expression levels of miR- 1, -30, -128, and -296 were observed in HCV patients compared to healthy controls, no significant differences were found between responders and non-responders following the first peg-IFN injection. The authors suggested that the findings might implicate that IFN treatment as well as HCV infection exerted a more profound effect on the liver rather than the PBMCs.

Although miRNA profiling of tissues from HCV infected individuals has shed considerable light on the relevance of miRNA modulation in disease pathogenesis, several extraneous factors including medications, age, and other factors may contribute to the observed changes. From that viewpoint, *in vitro* systems of HCV infection are likely to provide valuable information on the direct effect of HCV infection on cellular miRNA levels and its contribution to the development of disease. Bandyopadhyay and colleagues reported a significant down-regulation of miR-29 in hepatoma cells infected by cell culture-derived HCV as well as in liver samples of chronic HCV infected patients [70]. Interestingly, miR-29 over-expression in the liver cells led to a considerable decrease in HCV RNA indicating an antiviral effect of the miRNA. Similarly, Banaudha *et al.,* [66], using an in vitro approach to infect primary hepatocytes with HCV, observed an up-regulation of miRNAs (miR- 141 and -200) that target the DLC-1 tumor suppressor gene, which is frequently deleted in hepatocellular carcinoma. Artificial depletion of miR-141 led to the inhibition of HCV replication and virion production. These data suggest that HCV may have the ability to induce cellular miRNA changes that positively regulate the viral lifecycle in the infected cell.

Several HCV proteins have been implicated in interacting with components of the RNAi machinery to prevent the viral genome from being a target of the RNAi. The IRES and replicative intermediate of HCV, both being double stranded RNA structures have been shown to be substrates for Dicer [111]. However, over-expression of the core protein of HCV can abrogate the function of the RNase III enzyme to process precursor dsRNAs into siRNAs through direct interaction between the N terminal domain of the viral protein with Dicer [111,112]. The E2 structural protein of HCV has also been implicated in inhibiting siRNA mediated gene silencing by binding to Ago-2 [79]. Although it appears that specific HCV proteins possess the ability to suppress RNAi mediated silencing, the relevance of these interactions in the context of a viral infection is yet to be explored. Recent data also suggest the potential role of HCV proteins in modulating the expression of cellular factors through miRNAs. For example, the *in vitro* expression of the HCV genotype 3a core protein (but not 1b), which leads to the appearance of large lipid droplets, down-regulated tensin homolog deleted on chromosome 10 (PTEN) expression by a mechanism involving a microRNA-dependent blockade of PTEN translation [113]. As steatosis is frequently observed in patients infected with HCV, this study suggests that viral proteins may be directly involved in the deregulation of the lipid metabolism through miRNAs in the HCV-infected liver.

## 3. Potential Role of miRNAs in HCV/HIV-1 Co-Infection

The HIV-1 life cycle is fundamentally different from that of HCV. HIV-1 is a retrovirus that enters susceptible host cells such as CD4+ T cells and macrophages containing two copies of positive sense, single-stranded RNA (which encodes for nine genes) and enzymes needed for its productive infection such as reverse transcriptase, protease and integrase. The viral RNA undergoes reverse transcription to form double stranded viral DNA, which is transported to the nucleus for integration into the host genome [114]. On the other hand, HCV replicates via an RNA-dependent RNA polymerase and does not have a DNA intermediate [49]. Despite their well-characterized differences at the molecular biology level, the pathogenesis of HCV and HIV-1 co-infection remains poorly understood.

The development of HCV infection is dramatically influenced during co-infection with HIV-1, resulting in augmented viral replication and accelerated progression to chronic hepatitis [115]. About 30% of HIV infected individuals are estimated to be co-infected with HCV [116]. With the development of the current regimens of anti-retroviral therapy (cART), life expectancy of HIV-1 infected individuals has extended significantly. Cirrhosis and end-stage liver disease has become one of the predominant causes of morbidity and mortality in HIV-1/HCV co-infected patients on cART, accounting for up to 50% of all deaths [117,118]. In addition, it has been characterized that patients with HIV-1/HCV co-infection have higher HCV RNA abundance as compared to HCV mono-infected patients [119,120], insinuating potential molecular mechanisms that may be altered in the co-infected patients.

The mechanisms underlying the mutually influential roles of HIV-1 and HCV in co-infected patients are still incompletely understood. Some studies have reported that HCV may negatively affect proliferation of T cells by up-regulating apoptotic pathways [121,122]. On the other hand, HIV-1 infection of CD4+ T cells has a profound effect in impairing adaptive immune responses towards HCV infected cells contributing to poor natural clearance of the virus [123]. A recent study has suggested that the chronic immune activation characteristic of HIV-1 infection and progression to AIDS [124,125] is a distinguishing feature of the host response to HCV/HIV-1 co-infection [126].

Previous studies have demonstrated that HCV mono- and HCV/HIV-1 co-infection induce unique immunologic gene expression profiles in PBMCs [127]. While HIV-1 has evolved to successfully infect cells of the monocyte/macrophages lineage [128], productive infection of HCV in these cells is controversial [129]. However, preliminary evidence of potential HCV replication in monocytes and macrophages has been reported but it requires substantial exploration [130,131]. Despite numerous reports in addition to the ones mentioned above that have studied HIV-1/HCV co-infection, very few have characterized specific alterations at a molecular level that could play a crucial role in HIV-1/HCV co-infected patients.

The role of miRNAs in HIV-1 infection is a vastly expanding field [132,133,134]. The importance of microRNA modulation in HIV-1 replication has been reported, albeit only in the context of HIV-1 mono-infection. In a seminal paper in 2007, Huang *et al.,* found that the 3' ends of HIV-1 messenger RNAs are targeted by a cluster of cellular miRNAs including miR-28, miR-125b, miR-150, miR-223 and miR-382, which are enriched in resting CD4+ T cells as compared to activated CD4+ T cells [135]. Their data demonstrates that cellular miRNAs are pivotal in HIV-1 latency and suggests that manipulation of cellular miRNAs could be a novel approach for purging the HIV-1 reservoir. In addition, a potential role for miR-28, miR-150, miR-223 and miR-382 as “anti-HIV-1 miRNAs” has been recently reported in monocytes [136]. The authors suggested that the existence of these anti-HIV miRNAs contributed to the low susceptibility of monocytes to HIV-1, and differentiation of monocytes into macrophages reduced these miRNAs facilitating infection and active replication [136]. However, their role as anti-HIV-1 miRNAs in monocytes and their modulation during monocyte-to-macrophage differentiation remains highly controversial issues [137]. Likewise, miR-198 and miR-27b in monocytes and CD4+ T cells, respectively, have been shown to inhibit HIV-1 replication by repression of Cyclin T1 expression, an important cellular factor required for Tat-mediated transactivation of HIV-1 LTR-directed gene expression [138,139]. In addition to the cellular miRNAs, HIV-1’s own TAR miRNA is encoded in the viral LTR and manipulating this miRNA through drugs has been shown to reduce viral transcription (19). Overall, these reports emphasize the key role of miRNAs in suppressing HIV-1 infections.

Given the plethora of publications characterizing the importance of various microRNAs in HIV-1 and HCV infection independently, it is surprising that no reports thus far have characterized the alterations in microRNA expression profiles upon HIV-1/HCV co-infection *in vitro* or in patients. Based on data in the literature, we have identified several miRNAs that could be of relevance for the pathogenesis of HCV/HIV-1 co-infection.

- *MicroRNA-122*. As mentioned above, miRNA-122 plays a critical role in HCV life cycle [29,30,60,140]. Interestingly, HIV-1 infection of T cell lines, significantly up-regulated the expression of miR-122 [141]. Of note, miR-122 is undetectable in uninfected, quiescent T cells [142], and although miR-122 is detected in human primary macrophages, its expression levels are significantly lower when compared to a human hepatoma cell line (Huh7.5) (Swaminathan, G. et al., unpublished data). Would an increase in miRNA-122 expression upon HIV-1 infection of CD4+ T cells, monocytes and macrophages explain the ability of HCV to infect cells beyond hepatocytes in co-infected patients?
- *MicroRNA-29. *As previously mentioned, miR-29 (miR 29a, b and c) has been shown to be down-regulated in hepatocytes upon HCV infection, and miR-29 over-expression significantly reduced HCV replication [70]. Interestingly, miR-29a was reported to bind HIV-1 3’UTR by base-pair complementarities and target the viral RNA to P-bodies for degradation [71]. Another independent report characterized that ectopic expression of miR-29a inhibits HIV-1 protein Nef, in addition to reducing viral infectivity [143]. Thus, it would be very interesting to explore the modulation of miR-29 upon HIV-1/HCV co-infection and to determine if over-expression of miR-29 could have dual-inhibitory activity in HIV-1/HCV co-infected cells.
- *MicroRNA-149.* miR-149 was shown to be up-regulated by about 10-fold upon HCV infection [88]. Intriguingly, miR-149 was predicted to bind to HIV-1 3’-UTR and to the HIV-1 protein Vpr [144]. In addition, HIV-1’s own miRNA denoted as hiv1-miR-H1 has been shown to down regulate the expression of cellular miR-149 through direct interaction [145].
- *MicroRNA-199a*. The role of miR-199a-3p in liver diseases remains controversial. MiR-199a-3p has been associated with progression to liver fibrosis [146] and other liver injuries [147]. However, other studies have found down-regulated expression of miR-199a/b in the majority of HCCs from their cohorts [148]. Interestingly, while over-expression of miR-199a was shown to inhibit HCV replication by binding to the viral 5’UTR [54], it was also reported that HIV-1 infection resulted in greater than two-fold increase in miR199a levels [149]. Based on these data, it would be interesting to explore the potential role of miR-199a in HCV/HIV-1 co-infection.
- *MicroRNA-223*. This “anti-HIV-1” microRNA was validated to inhibit HIV-1 replication by binding to HIV-1 3’UTR by several groups [135,136]. It is worth noting that miR-223 was found to be down-regulated by about 4.8-fold in HCV-induced hepatocellular carcinoma from HCV infected patients [95]. However, it has not been investigated whether miR-223 expression is down-regulated in the liver of HCV/HIV-1 co-infected patients with HCC, and if so, whether it would correlate with higher HIV-1 viral load.
- *Let7 microRNAs*. Let 7b and Let 7g microRNAs were shown to be significantly decreased in PBMCs and CD4+ T cells of HIV-1 infected patients as compared to healthy controls or patients who can naturally control HIV-1 infection (Long Term Non-Progressors, LTNP; and elite suppressors) [150]. Very recently, Let7b has been shown to bind to HCV protein NS5B and to the 5’UTR, and to significantly suppress HCV infection [55]. In a scenario where HIV-1 replication can suppress Let7b levels, it is plausible that a decrease in Let7b miRNA in the context of co-infection could potentially augment HCV replication.

## 4. Conclusions and Perspectives

The role of miRNAs in regulating not only normal cellular processes but also their contribution to viral pathogenesis and the replication of viruses is already remarkable, despite the fact that we may have uncovered only a fraction of the likely very large number of functional interactions of microRNAs with implications in viral pathogenesis. HCV in particular appears to be profoundly influenced by the host intracellular miRNA environment for its replicative cycle. The ability of the virus to hijack the cellular miRNA machinery for its survival serves as yet another example of how viruses co-evolve with the host. HCV infection is associated with intracellular miRNA changes that are induced as a combined consequence of the host response and perhaps direct effects of the virus. These miRNA alterations have been proposed to serve as useful biomarkers for diagnosis, prognosis and response to therapy during HCV infection. Furthermore, over the past few years, the concept of extracellular, circulating miRNAs has gained momentum for its potential to serve as non-invasive molecular markers for various diseased states [73,151,152]. Circulating miRNAs shown to exist in two forms—as exosomes/microvesicles encapsulated and as Ago2-associated miRNAs that are highly stable and resistant to nuclease degradation [153,154,155]. One of the mechanisms for release of miRNAs into the circulation is from cells undergoing necrosis and apoptosis. In regard to HCV infection, serum miR-122 is found to be elevated in chronically HCV infected patients likely due to hepatic tissue damage and it correlates with levels of serum alanine aminotransferase and aspartate aminotransferase, and with the histologic activity index score in the liver [91]. In contrast, other studies have found no correlation of miR-122 expression of liver biopsies with viral load from subjects with chronic hepatitis C undergoing IFN therapy; and markedly decreased pretreatment miR-122 levels in subjects who had no virological response during later IFN therapy [99]. Therefore, the potential use of miR-122 as a biomarker in chronic hepatitis C and HCC needs to be further defined and remains as a possibility that requires further investigation. Apart from miR-122, HCV patients also exhibit increased serum levels of miR-34a and -16 wherein both miR-122 and -34a are reflective of the liver fibrotic stage and hepatic inflammation [92]. In-depth knowledge of miRNA modulation in HCV associated disease will aid in truly exploiting the potential of miRNAs to serve as biomarkers with a real diagnostic or prognostic value.

Apart from the biomarker purpose, identification of miRNA alterations that make the cellular environment more conducive to HCV replication could reveal targets for antagomir-based therapy against HCV infection. Indeed, mounting knowledge on the role of cellular miR-122 in maintaining HCV abundance has ultimately led to the development of the first antagomir based therapeutic intervention against HCV infection. The advancement of Miravirsen to phase 2a clinical trials [38] represents not only a milestone for the field of HCV research but also for the antimiR-based technology as a promising therapeutic platform. However, caution needs to be maintained, as it remains unknown if long-term suppression of an endogenous miRNA will adversely affect the homeostatic balance maintained by the body. 

A current challenge is to get a better molecular understanding of HCV/HIV-1 co-infection. This knowledge is essential to clearly comprehend the differential molecular mechanisms that may contribute to pathogenesis in infected patients. The expression of miRNAs is highly specific to the cell type or tissues being studied. A difficulty towards the understanding of the role of the miRNAome in HCV/HIV-1 co-infection is to identify cell types with biological significance for both viruses to perform mechanistic studies in cell culture. It is important to point out that most of the studies in the literature characterizing microRNA changes upon HCV infection are in hepatoma cell lines or liver tissue from HCV-infected patients. However, the liver is neither targeted by HIV-1 to establish a productive infection nor has a major role in the development of AIDS. In addition, the ability of HCV to productively infect human cells other than hepatocytes is controversial. In spite of these major differences in tropism, both viruses influence the complex pathogenesis in HCV/HIV-1 co-infected individuals, albeit to varying extents. A recent report characterized microRNA changes in PBMCs from HIV-1 infected patients [156]. These authors noted that 33% of global microRNAs that were up-regulated by HCV in hepatoma cell lines, as reported by another group [88], were also up-regulated in PBMCs from HIV-1 infected patients in their study. These data suggests that host cells might respond to pathogens using similar miRNAs as part of the interactions between the pathogen and the host. Conversely, there was no overlap in the miRNAs down-regulated by each virus, which indicates that viruses might target certain miRNAs as part of a general virus-host interaction to aid replication and survival.

Emerging evidence demonstrates that secreted miRNAs are key regulators of the tumor microenvironment in the liver, and neurodegenaration in the central nervous system [157,158]. The recent discovery of miRNAs as paracrine agonists of TLR7 and TLR8 [157] could represent new avenues for the treatment of chronic viral infections, such as HCV and HIV-1. Furthermore, although the role of exosomes in cell-to-cell communication is well known (reviewed in [159]), several groups have recently demonstrated that secreted miRNAs enclosed in exosomes have an important role in cell-to-cell communication (reviewed in [160]). In particular, the functional transfer of miRNAs between cells of the same or distinct type has been reported [161,162,163]. Indeed, there are significant recent advances on the intercellular transfer of miRNAs by exosomes in several clinically relevant scenarios, such as cancer [163,164,165,166], the regulation of the immune system [167,168], and liver [169,170], central nervous system [171], and infectious diseases [172]. To add to this intriguing interplay between exosome-mediated cell-to-cell communication and horizontal modulation of gene expression by miRNAs, some viruses including HIV-1 and HCV, utilize exosomes for intercellular communication [173,174,175,176], and viruses that express miRNAs exploit the endosomal-exosomal pathway for intercellular cross-talk and immune evasion [177]. Thus, considering the different tropism of HIV-1 and HCV viruses, it is possible that circulating miRNAs in exosomes secreted by non-hepatocyte HIV-1-infected cells were taken by HCV-infected hepatocytes as a part of the cell-to-cell communication process, and affect the expression of some cellular factors in the HCV-infected hepatocyte which may ultimately impact disease outcome in HCV/HIV-1 co-infected patients.

Overall, the modulation of the expression of miRNAs in the human population represents a difficult task in the years to come. Could it be possible in the future to successfully fight against viral infections using “antagomir” or “mimics” as therapeutic approaches with none or minimal side effects? The challenge is on us.
